# Crystalloid vs colloid fluid resuscitation in polytrauma patients: a systematic review

**DOI:** 10.1007/s00068-025-02921-8

**Published:** 2025-08-11

**Authors:** Julia Dormann, Karolina Dahms, Eva Steinfeld, Kelly Ansems, Heidrun Janka, Maria-Inti Metzendorf, Thomas Breuer, Klemens Horst, Frank Hildebrand, Gernot Marx, Carina Benstoem

**Affiliations:** 1 Department of Intensive Care Medicine and Intermediate Care, Medical Faculty, RWTH Aachen University, Pauwelsstr. 30, 52074 Aachen, Germany; 2 Institute of General Practice, Medical Faculty of the Heinrich-Heine-University Dusseldorf, Dusseldorf, Germany; 3 Department of Orthopaedics, Trauma and Reconstructive Surgery, Medical Faculty, RWTH Aachen University, Aachen, Germany

**Keywords:** Polytrauma, Colloid, Crystalloid, Intensive care medicine

## Abstract

**Purpose:**

The question of whether crystalloid or colloid fluid yields better outcomes in the treatment of polytrauma patients has recently garnered significant interest. This systematic review aims to comprehensively compare the effects of crystalloid versus colloid fluid resuscitation on treatment outcomes in polytrauma patients.

**Methods:**

We searched PubMed, Cochrane Central Register of Controlled Trials and Web of Science to identify completed and ongoing studies from inception of each database to August 9, 2022. We included systematic reviews and randomized controlled trials (RCTs) comparing crystalloid versus colloid fluid resuscitation on treatment outcomes in polytrauma patients admitted to the intensive care unit (ICU).

**Results:**

We included one RCT with a total of 2,857 adult participants (mean age 63 years in the colloids and crystalloids group, 62.2% male in the colloids group and 62.5% male in the crystalloids group). Our findings indicate that the administration of crystalloids does not significantly differ from colloids in terms of 28-day mortality among polytrauma patients. (HR 1.19; 95% CI 0.54 to 2.62; RD 23 more per 1000, 95% CI 58 fewer to 174 more, 1 study, 177 participants, very low quality of evidence).

**Conclusion:**

Crystalloid volume therapy has no significant difference on adult polytrauma patients compared to colloid volume therapy regarding mortality.

## Introduction

Polytrauma, characterized by the simultaneous occurrence of multiple traumatic injuries in an individual, presents a significant challenge to healthcare providers and remains a leading cause of morbidity and mortality worldwide [1]. The importance of rapid, efficient and goal-oriented volume therapy in the management of polytrauma patients is indisputable [2]. For most patients, appropriate volume therapy can maintain or restore adequate plasma volume and cardiac preload, thereby ensuring or improving organ perfusion and microcirculation [3]. The management of fluid and volume therapy requires a precise balance to avoid the risks of hypoperfusion from insufficient volume administration or the development of dilutional coagulopathy and increased bleeding due to excessive fluid intake [4].

The principal aim of volume therapy is to augment cardiac output and guarantee sufficient oxygen delivery [5]. A critical decision lies in selecting between crystalloid and colloid fluids for intravascular volume therapy [6]. Nonetheless, determining the optimal fluid type for polytrauma patients continues to be a subject of ongoing debate within the medical community.

The inquiry of whether crystalloid or colloid fluid provides better outcomes in the treatment of polytrauma patients has attracted significant interest and research over time. Although crystalloids are widely accessible, affordable, and generally safe, colloids possess potentially advantageous characteristics, including expansion of intravascular volume and sustained hemodynamic stability [6]. Nonetheless, worries concerning the safety and possible unfavorable effects of colloids, such as coagulopathy and renal dysfunction, remain [7].

To address clinical uncertainties and provide evidence-based guidance, this systematic review aims to compare comprehensively the effect of crystalloid versus colloid fluid resuscitation on treatment outcomes in polytrauma patients admitted to the intensive care unit (ICU).

## Methods

This review is part of the guideline project ‘S3-Leitlinie Intensivmedizin nach Polytrauma’ (AWMF Nr. 040—014) guided by the German Interdisciplinary Association of Critical Care and Emergency Medicine (Deutsche Interdisziplinäre Vereinigung für Intensiv- und Notfallmedizin, DIVI) and the German Society for Anaesthesiology and Intensive Care Medicine (Deutsche Gesellschaft für Anästhesiologie und Intensivmedizin, DGAI). The aim was to summarize the current evidence in the field of polytrauma to formulate specific recommendations. All studies that were carried out as part of this project used the same methodology which was consented within the guideline group.

### Eligibility criteria

We included studies comparing crystalloid volume therapy compared to colloid volume therapy in adult polytrauma patients admitted to the ICU that met the following inclusion criteria:

- Age of the included patients is ≥ 18 years
- Polytrauma present and defined as: a simultaneous injury to multiple body regions or organ systems, at least one or more of which, in combination, is life-threatening [1]
- Randomized controlled trial (RCT) or systematic review that includes RCTs
- Language of publication: English or German
- No multiple publication without additional information
- Publication accessible as full text
- Comparison of crystalloid volume therapy and colloid volume therapy

### Search strategy

We conducted a systematic search in the following sources from inception of each database to August 9, 2022 with no restrictions on the language of publication:

- MEDLINE (PubMed)
- Cochrane Central Register of Controlled Trials (CENTRAL) via Cochrane Register of Studies Online
- Web of Science (Science Citation Index and Emerging Citation Index)

Details of our search strategy are provided in the Appendix No 1. In addition, we searched reference lists of included studies to identify other potentially eligible studies.

### Study selection

We imported citations from the systematic search into the Rayyan Systematic Review App (Rayyan, Cambridge, MA, USA) [8]. Three authors independently screened the titles and abstracts of all potential studies. Full-text study publications were retrieved, imported into Microsoft Excel (Microsoft, Redmond, WA, USA) and screened by two authors independently. Reasons for exclusion of ineligible studies were recorded (Appendix No 2). Any disagreements were resolved through discussion or, if required, consultation with a third author.

### Data collection process

We used a customised data collection form developed in Microsoft Excel to collect study data [9]. The following data were obtained:

1. Study characteristics: authors, publication date, and study design
2. Participants characteristics: number of included participants, gender, age
3. Intervention: intervention, control
4. Clinical outcomes: all-cause mortality (day 28, day 60, time to-event, and up to longest follow-up), clinical status, serious adverse events (SAE), adverse events (AE), infections, quality of life, length of stay.

Extraction of study characteristics and outcome data of included studies was conducted by one author and checked by another. Any disagreements were resolved by discussion or by consulting a third review author if necessary. Two authors transmitted the outcome data into a statistical software (RevMan 5.3, Cochrane, London, England), which was checked by a third author [10]. Missing data resulted in the exclusion of the study in the analyses of the missing outcome.

### Study risk of bias assessment

Two authors independently assessed the risk of bias of included studies using the Risk of Bias 2 (RoB 2) tool (Cochrane, London, England) [11]. RoB 2 addresses five domains of bias (randomisation process, deviations from intended interventions, missing outcome data, measurement of the outcome, selection of the reported results). The signalling questions recommended in the tool were used to make a judgement according to the available options. Algorithms proposed in RoB 2 were used to assign each domain and the overall risk of bias, a level of bias (low risk of bias, some concerns, high risk of bias). We resolved any disagreements by discussion or by involvement of another author.

### Synthesis methods

To summarize demographics, we used descriptive statistics. A meta-analysis was performed only, if the clinical and methodological characteristics of individual studies were sufficiently homogeneous. For all analyses, we used Rev-Man 5.3 [10]. Data entry into the Rev-Man software was checked by a second review author for accuracy. Outcome data were pooled using the random-effects model, as we anticipated that true effects would be related, but not the same for the studies included in our review. For dichotomous data, we performed analyses using the Mantel–Haenszel method under a random-effects model to report pooled risk ratios (RR) with 95% confidence intervals (CI). For continuous outcomes, we calculated mean differences with 95% CIs. Forest plots were provided to summarize the effects from individual studies. When data was lacking or incomplete for analysis, such information was reported narratively. A *p*-value of < 0.05 was considered as statistically significant. The data underwent analysis utilizing the Cochrane methodology.

### Certainty assessment

We used used GRADEpro Guideline Development Tool Software (McMaster University and Evidence Prime Inc., Hamilton, Ontario, Canada) to create a summary of findings table and evaluated the certainty of the evidence using the GRADE approach for interventions evaluated in RCTs [12].

## Results

**Fig. 1:**
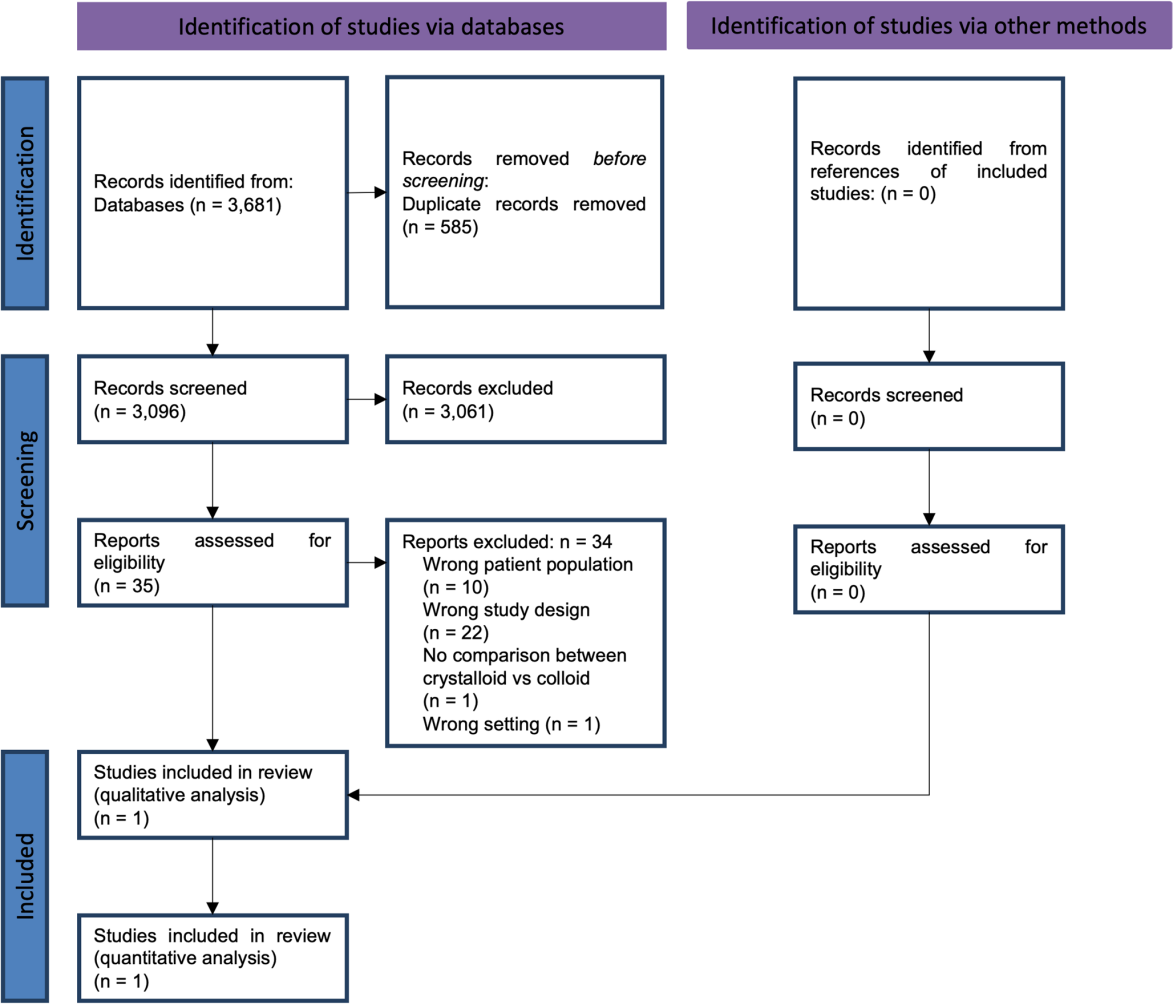
Flowchart of the systematic review selection process

The search identified 3,681 records. After removing duplicates, we screened 3,098 records based on title and abstract, of which 3,061 did not meet the prespecified inclusion criteria and were excluded. We screened the full texts of the remaining 35 references. No additional full texts were identified. 34 records were excluded for different reasons (Fig. 1). One RCT was included in our meta-analysis: Annane et al.[13].

### Study characteristics

One RCT [13] with a total of 2,857 adult participants (mean age 63 years in the colloids and crystalloids group, 62.2% male in the colloids group and 62.5% male in the crystalloids group) is included. The included RCT used parallel-group design. The randomization was stratified by center and by three admission diagnoses: sepsis, multiple trauma, or other causes. In the context of our research question, we were only interested in the multiple trauma group. Additional study characteristics are provided in Table 1.

**Table 1:** Study characteristics

Study (year)	Study design	No. of patients	Median age (IQR) in years	Gender (m/f)	Intervention	Control
Annane et al [13]	RCT	*N* = 2857 Colloids Group Total: *N* = 1414 Trauma: *N* = 85 Crystalloids Group Total: *N* = 1443 Trauma: *N* = 92	Colloids group 63 (50–76) Crystalloids Group 63 (50–75)	Colloids group 880/534 Crystalloids Group 902/541	Colloids group: allowed treatments included hypooncotic (eg, gelatins, 4% or 5% of albumin) and hyperoncotic (eg, dextrans, hydroxyethyl starches, and 20% or 25% of albumin)	Crystalloids group: allowed treatments included isotonic or hypertonic saline and any buffered solutions

*IQR* interquartile range

### Results of individual studies

Crystalloid volume therapy compared to colloid volume therapy in adult polytrauma patients (Table 2).

**Table 2:** Summary of findings table

Outcome	No. of participants (studies) follow Up	Certainty of the evidence (GRADE)	Relative effect (95% CI)	Anticipated absolute effect	
				**Risk with crystalloids**	**Risk difference with colloids**
28-day mortality	177 (1 RCT)	** ⊕ ◯◯◯** Very low ^a,b^	**HR 1.19** (0.54 to 2.60)	130 per 1000	**23 more per 1000** (58 fewer to 174 more)

*The risk in the intervention group (and its 95% confidence interval) is based on the assumed risk in the comparison group and the relative effect of the intervention (and its 95% CI)

*CI* confidence interval, *HR* hazard ratio

a. Colloids used in the crystalloid group and crystalloids used in the colloid group

b. Due to 95% confidence interval including both benefits and harms; few participants; only one study

### 28-day mortality

**Fig. 2:**
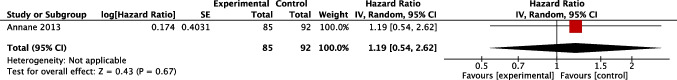
Forest plot describing the difference between administration of colloids compared to crystalloids regarding all-cause mortality at day 28

The study reported all-cause mortality at up to day 28 for 177 participants (Fig. 2) and indicated that the administration of crystalloids did not significantly differ from colloids in terms of 28-day mortality among polytrauma patients. (hazard ratio (HR) 1.19; 95% CI 0.54 to 2.62; certainty of evidence very low) [13].

No results were reported for all-cause mortality (day 60, time to-event, and up to longest follow-up), clinical status, serious adverse events (SAE), adverse events (AE), infections, quality of life and length of stay.

### Methodological appraisal

The overall risk of bias was low for all outcomes.

## Discussion

The present study, involving over 170 participants, demonstrated no significant difference in 28-day mortality when using crystalloid or colloid fluids for adult polytrauma patients in the intensive care unit. However, these findings must be contextualized within the broader landscape of fluid management strategies for critically ill patients.

The S3 guideline “Polytrauma/Serious Injury Treatment” provides explicit recommendations for volume resuscitation strategies in polytrauma patients [14]. The guideline suggests the use of crystalloid volume substitutes as the preferred option, while isotonic saline solutions should be avoided. If crystalloid whole electrolyte solutions fail to stabilize circulation, the guideline suggests considering colloidal whole electrolyte solutions (such as gelatin preparations and human albumin) for hypotensive polytrauma patients. However, this guideline addresses only the treatment phase leading up to initial surgical care and does not address subsequent treatment in the ICU.

Applying established guidelines and recommendations, like the S3 Guideline “Intravascular Volume Therapy in Adults” [15] or “Recommendations for hemodynamic monitoring in internal medicine intensive care” [16], from broader contexts can offer useful guidance, but it is crucial to appreciate the necessity of patient-specific considerations and adaptations in the absence of dedicated polytrauma-focused research.

A meta-analysis by Safiejko et al., encompassing 28 randomized controlled trials and 4,503 patients with traumatic hemorrhagic shock, revealed that hypotensive fluid resuscitation resulted in reduced mortality rates, fewer adverse events, and a lower incidence of conditions such as fever, acute respiratory distress syndrome, and multiple organ dysfunction syndrome. However, hypovolemic fluid therapy was associated with complications including thrombocytopenia, renal failure and anaemia [17].

Another meta-analysis on permissive hypotension by Tran et al. in trauma patients with hemorrhagic shock included five randomized studies with a total of 1,158 patients. The intervention arms aimed for blood pressure targets ranging from a systolic blood pressure of 50 mmHg to 70 mmHg or a mean arterial pressure of 50 mmHg or higher, compared to the control group’s targets of a systolic blood pressure of 65 mmHg to 100 mmHg or a mean arterial pressure of 65 mmHg or higher. The pooled odds ratio (OR) of 0.70 (95% CI, 0.53–0.92) indicated a survival advantage with permissive hypotension. These patients required fewer blood products and experienced less blood loss. Nevertheless, limited statistical power and the absence of blinding and protocol presentation in three studies led to inconclusive outcomes [18].

In a subsequent meta-analysis by Owattanapanich et al., the efficacy of hypotensive fluid and volume therapy was examined in individuals experiencing traumatic hemorrhagic shock, focusing on mortality, acute respiratory distress syndrome (ARDS), acute kidney injury, and multiple organ dysfunction. This analysis encompassed 30 studies and indicated a statistically significant reduction in mortality, along with diminished requirements for fluid administration and transfusions. Furthermore, the therapy exhibited a safeguarding influence on multiple organ dysfunction and the development of ARDS. However, no substantial divergence was observed concerning acute kidney failure.[19].

These investigations predominantly focus on the initial phase of treatment and do not explicitly address the complex therapeutic interventions administered in the intensive care unit. Furthermore, they do not encompass polytrauma patients, leaving the applicability of these findings to the intensive care of polytrauma patients uncertain. Nevertheless, there is a presumption that implementing a limited volume and fluid management approach may offer greater advantages to these patients.

A notable limitation of this study is the scarcity of randomized controlled trials, as only one RCT was identified in this systematic review. Additionally, this single trial had a small sample size, which further limits the study's findings.

## Conclusion

In conclusion, managing volume therapy for polytrauma patients after immediate emergency care presents a challenging and complex aspect of critical care medicine, with a notable lack of specific evidence in the existing literature.

Hence, there is a need for more dedicated research in this area. The need for individualised approaches in the constantly evolving landscape of polytrauma care is underscored by the delicate balance between optimizing perfusion and avoiding microcirculatory disorder.

Crystalloid volume therapy has no significant difference on adult polytrauma patients compared to colloid volume therapy regarding mortality.

## Data Availability

Additional study data are available from the corresponding author.

## Appendix 1 Search strategy

**1. PubMed**

#1

"Multiple Trauma"[mh] OR"Trauma Centers"[mh] OR"Craniocerebral Trauma"[mh] OR"Critical Illness"[mh] OR"Critical Care"[mh:noexp] OR polytrauma*(20) OR"multiple trauma*"(20) OR multitrauma(20) OR"major trauma*"(20) OR"multiple injur*"(20) OR"multisystem trauma"(20) OR"multisystem injur*"(20) OR"multitrauma"(20) OR"trauma patient*"(20) OR"trauma population"(20) OR"trauma care"(20) OR"trauma cent*"(20) OR"trauma team*"(20) OR"critical illness*"(20) OR"critically ill"(20) OR"critical care"(20) OR"intensive care"(20) OR"cranial trauma*"[tiab] OR"traumatic brain injur*"(20) OR"craniocerebral trauma*"(20) OR"cranio-cerebral trauma*"(20) OR"traumatic encephalopathy*"(20)

#2

"Fluid Therapy"[mh] OR"Crystalloid Solutions"[mh] OR"Colloids"[mh] OR"Isotonic Solutions"[mh:noexp] OR"Rehydration Solutions"[mh] OR Tranexamic Acid[mh] OR"fluid therap*"(20) OR"fluid resuscitation*"(20) OR"hypertonic saline"(20) OR"tranexamic acid"(20) OR ("volume therap*"[tiab] AND (crystalloid*[tiab] OR colloid*[tiab]))

#3

#1 AND #2

#4

systematic [sb]

#5

#3 AND #4

 = 283 hits

#6 *[Cochrane RCT-Filter, sensitivity max. version]* [20]

(randomized controlled trial(21) OR controlled clinical trial(21) OR randomized[tiab] OR placebo[tiab] OR drug therapy(22) OR randomly[tiab] OR trial[tiab] OR groups[tiab]) NOT (animals[mh] NOT humans[mh])

#7

#3 AND #6

 = 884 hits

**2. CENTRAL (via Cochrane Register of Studies Online)**

#1 (fluid therap* OR crystalloid solution* OR colloids OR isotonic solution* OR rehydration solution* OR tranexamic acid* OR fluid resuscitation* OR hypertonic saline* OR tranexamic acid* OR (volume therap* AND (crystalloid* OR colloid*))):TI,AB,KY

#2 (multiple trauma* OR craniocerebral trauma* OR critical illness* OR critical care* OR polytrauma* OR multitrauma OR major trauma* OR multiple injur* OR multisystem trauma* OR multisystem injur* OR trauma patient* OR trauma population* OR trauma care* OR trauma cent* OR trauma team* OR critical illness* OR critically ill OR intensive care* OR cranial trauma* OR traumatic brain injur* OR craniocerebral trauma* OR cranio-cerebral trauma* OR traumatic encephalopathy*):TI,AB,KY

#3

#1 AND #2

 = 1360 hits

**3. Web of Science (Science Citation Index und Emerging Citation Index)**

1. (TI = ("Fluid Therap*"OR"Crystalloid Solutions"OR"Colloids"OR"Isotonic Solutions"OR"Rehydration Solutions"OR"fluid resuscitation*"OR"hypertonic saline"OR"tranexamic acid"OR ("volume therap*"AND (crystalloid* OR colloid*)))) OR AB = (("Fluid Therap*"OR"Crystalloid Solutions"OR"Colloids"OR"Isotonic Solutions"OR"Rehydration Solutions"OR"fluid resuscitation*"OR"hypertonic saline"OR"tranexamic acid"OR ("volume therap*"AND (crystalloid* OR colloid*))))

2. TI = (polytrauma* OR"multiple trauma*"OR multitrauma OR"major trauma*"OR"multiple injur*"OR"multisystem trauma"OR"multisystem injur*"OR"multitrauma"OR"trauma patient*"OR"trauma population"OR"trauma care"OR"trauma cent*"OR"trauma team*"OR"critical illness*"OR"critically ill"OR"critical care"OR"intensive care"OR"cranial trauma*"OR"traumatic brain injur*"OR"craniocerebral trauma*"OR"cranio-cerebral trauma*"OR"traumatic encephalopathy*") OR

AB=(polytrauma* OR"multiple trauma*"OR multitrauma OR"major trauma*"OR"multiple injur*"OR"multisystem trauma"OR"multisystem injur*"OR"multitrauma"OR"trauma patient*"OR"trauma population"OR"trauma care"OR"trauma cent*"OR"trauma team*"OR"critical illness*"OR"critically ill"OR"critical care"OR"intensive care"OR"cranial trauma*"OR"traumatic brain injur*"OR"craniocerebral trauma*"OR"cranio-cerebral trauma*"OR"traumatic encephalopathy*")

3. 1 AND 2

4. TI = (random* OR placebo OR trial OR groups) OR AB = (random* OR placebo OR trial OR groups)

5. 3 AND 4

 = 1437 hits

## Appendix 2 Records excluded during full-text screening

**Table:**

Author	Year	Title	Reason for exclusion in full-text screening
Akbari et al	2018	The effect of fibrinogen concentrate and fresh frozen plasma on the outcome of patients with acute traumatic coagulopathy: a quasi-experimental study	No comparison between crystalloid vs colloid
Allen et al	2016	Is Hydroxyethyl Starch Safe in Penetrating Trauma Patients?	Wrong study design
Angele et al	2008	Bench-to-bedside review: Latest results in hemorrhagic shock	Wrong study design
Argalious	2012	Colloid Update	Wrong patient population
Bellomo	2002	Fluid resuscitation: Colloids vs. crystalloids	Wrong study design
Bellomo	2015	A pilot, randomized, blinded, multi-centre, feasibility, safety and biochemical and physiological study of normal saline versus plasmalyte in intensive therapy	Wrong study design
Bellomo et al	2006	The effects of saline or albumin resuscitation on acid–base status and serum electrolytes	Wrong patient population
Berger et al	1994	Unknown trace-element administration in trauma and burn patients	Wrong study design
Bradley	2001	Crystalloid, colloid or small volume resuscitation?	Wrong study design
Bruells et al	2015	The role of colloids in intensive care medicine. Evidence instead of emotions	Wrong study design
Chappell et al	2021	Safety and efficacy of tetrastarches in surgery and trauma: a systematic review and meta-analysis of randomised controlled trials	Wrong study design
Choi et al	1999	Crystalloids vs. colloids in fluid resuscitation: A systematic review	Wrong patient population
Coppola et a	2014	Fluid resuscitation in trauma patients: what should we know?	Wrong study design
Driessen et al	2006	Fluid therapy for the traumatized patient	Wrong study design
DuBose et al	2010	Clinical Experience Using 5% Hypertonic Saline as a Safe Alternative Fluid for Use in Trauma	Wrong study design
Ertmer et al	2018	Fluid therapy and outcome: a prospective observational study in 65 German intensive care units between 2010 and 2011	Wrong study design
Evans	2015	Effects of fluid resuscitation with colloids vs. Crystalloids on mortality in critically ill patients presenting with hypovolemic shock	Wrong study design
Gonzales-Castro et al	2016	The SPLIT trial: Anything new in critical care fluid therapy?	Wrong patient population
Hamada et al	2015	European trauma guideline compliance assessment: the ETRAUSS study	Wrong study design
Hartog et al	2012	Volume replacement after trauma: an update	Wrong study design
Holcomb	2010	Optimal Use of Blood Products in Severely Injured Trauma Patients	Wrong study design
Hwang et al	2018	Implementation of Trauma Center and Massive Transfusion Protocol Improves Outcomes for Major Trauma Patients: A Study at a Single Institution in Korea	Wrong study design
James et al	2011	Resuscitation with hydroxyethyl starch improves renal function and lactate clearance in penetrating trauma in a randomized controlled study: the FIRST trial (Fluids in Resuscitation of Severe Trauma)	Wrong patient population
Kramer	2003	Hypertonic resuscitation: Physiologic mechanisms and recommendations for trauma care	Wrong study design
Krishafor et al	2018	Comparative characteristics of liberal and restrictive fluid resuscitation in multiple trauma	Wrong study design
Kruer et al	2012	Colloids in the intensive care unit	Wrong study design
Orbegozo Cortés et al	2015	Crystalloids versus colloids: exploring differences in fluid requirements by systematic review and meta-regression	Wrong patient population
Perel et al	2013	Colloids versus crystalloids for fluid resuscitation in critically ill patients	Wrong patient population
Qureshi et al	2016	Meta-analysis of colloids versus crystalloids in critically ill, trauma and surgical patients	Wrong patient population
Ramesh et al	2019	Fluid resuscitation in trauma: what are the best strategies and fluids?	Wrong setting
Ripolles et al	2015	Colloids versus crystalloids in objective-guided fluid therapy, systematic review and meta-analysis. Too early or too late to draw conclusions	Wrong patient population
Rizoli	2003	Crystalloids and colloids in trauma resuscitation: A brief overview of the current debate	Wrong study design
Tseng et al	2020	Resuscitation fluid types in sepsis, surgical, and trauma patients: a systematic review and sequential network meta-analyses	Wrong patient population
York et al	2000	Fluid resuscitation of patients with multiple injuries and severe closed head injury: experience with an aggressive fluid resuscitation strategy	Wrong study design

## Abbreviations

AE: Adverse event
CI: Confidence intervals
DGAI: German Society for Anaesthesiology and Intensive Care Medicine (Deutsche Gesellschaft für Anästhesiologie und Intensivmedizin)
DIVI: German Interdisciplinary Association of Critical Care and Emergency Medicine (Deutsche Interdisziplinäre Vereinigung für Intensiv- und Notfallmedizin)
HR: Hazard ratio
ICU: Intensive care unit
IQR: Interquartile range
RCT: Randomized controlled trial
RD: Risk difference
RR: Risk ratio
SAE: Serious adverse event
